# Endoscopic transorbital approach to posterior fossa recurrent craniopharyngioma

**DOI:** 10.3171/2025.1.FOCVID24177

**Published:** 2025-04-01

**Authors:** Francesco Paglia, Ben Chat-Fong Ng, Xin-Liang Xu, Lorenzo Sgarbanti, Francesco Corrivetti, Matteo de Notaris, Hunter Kwok-Lai Yuen, Calvin Hoi-Kwan Mak

**Affiliations:** 1 Human Neurosciences Department, University La Sapienza, Rome, Italy;; 2 Department of Neurosurgery, Queen Elizabeth Hospital, Hong Kong;; 3 Department of Neurosurgery, University of Ferrara, Italy;; 4 Laboratory of Neuroanatomy, EBRIS Foundation, Salerno, Italy; and; 5 Hong Kong Eye Hospital Department of Ophthalmology and Visual Sciences, The Chinese University of Hong Kong, Hong Kong

**Keywords:** transorbital endoscopic approach, sagittal crest, meningo-orbital band, trigeminal porus, prepontine dura

## Abstract

Recurrent craniopharyngiomas represent a complex neurosurgical challenge. These histologically benign lesions show aggressive behavior that frequently recurs. The aim in treatment is to achieve an optimal balance between local control and quality of life. However, no agreement on the management of recurrent cases exists. Different surgical strategies are utilized for the management of these lesions using transcranial or endoscopic approaches. The authors present the case of a 38-year-old man with a large recurrent craniopharyngioma, previously treated with several endonasal interventions, responsible for headaches and unsteady gait. The recurrent cystic portion of the tumor, invading the pons, was treated using an endoscopic transorbital approach.

The video can be found here: https://stream.cadmore.media/r10.3171/2025.1.FOCVID24177

## Transcript

This video shows an endoscopic transorbital approach for resection of the cystic portion of a recurrent craniopharyngioma extending at the level of the brainstem.

### 0:29 Clinical Presentation and Neurological Examination.

The patient, a 38-year-old man suffering from a recurrent craniopharyngioma, previously treated with multiple endonasal surgery, transcranial operations, ventriculoperitoneal shunt, and Gamma Knife radiosurgery, was admitted for recurrent falls for a few weeks and became chairbound.

### 0:45 Neuroimaging and Choice of Approach.

The MRI showed a large recurrent craniopharyngioma with a cystic component extending at the level of the posterior cranial fossa and compressing the brainstem.

T2-weighted images showed the hyperintensity signal at the level of the pons, suggestive of brainstem edema. The aim of the surgery was to decompress the brainstem, and after discussion with the patient, endoscopic transorbital approach was chosen as the surgical approach. The endoscopic transorbital approach was chosen for different reasons.^1^ Firstly, a further endonasal approach may be associated with an increased risk of cerebrospinal fluid leak and infection. Secondly, a more invasive transcranial approach would not have guaranteed complete resection of the lesion, exposing the patient to an increased risk of perioperative complications.^2^ Anatomically, the transorbital approach provides a good trajectory to the anterolateral part of the pons, avoiding the sellar compartment and neurovascular structure, using a lateromedial and inferior-superior trajectory.

### 1:45 Positioning and Surgical Equipment.

The patient was put in the supine neutral position, with the head fixed in a three-pin skull clamp.

Intraoperative neuromonitoring was used, including brainstem auditory evoked response (BAER) and motor evoked potential (MEP). Abdominal fat was prepared for closure. Magnetic navigation with neuronavigated suction was used. Exposing the branches of the trigeminal nerve using an interdural dissection allows to expose and reach the prepontine dura mater rapidly, avoiding the need of further osteotomies.

### 2:00 Skin Incision.

A subbrow incision was made until the supraorbital ridge is reached and periorbita was dissected subperiostally.

The orbital rim was flattened using a coarse diamond burr, taking care to protect periorbita with a malleable retractor.

### 2:15 Subperiosteal Dissection of the Periorbita and Meningo-Orbital Band (MOB).

Periorbita was detached from the orbital bones using a subperiosteal technique until the meningo-orbital band had been reached.^3^

### 2:24 Drilling of the Greater Sphenoid Wing and Middle Fossa Exposure.

At this point, the greater sphenoid wing was drilled in order to identify middle fossa dura and sagittal crest,^4^ a landmark that has been described in endoscopic transorbital approaches in the literature. Sagittal crest was removed at this point.

Dissection of the MOB unlocked the dura away from the periorbita and enabled to start the interdural dissection of the middle cranial fossa floor, exposing the mandibular branch of the trigeminal nerve.

### 2:58 Anatomical Illustration of the Trigeminal Nerve and Its Branches.

This anatomical dissection shows the trajectory used to identify and open the trigeminal porus following the course of V3.

### 3:07 Opening of the Trigeminal Porus.

At this point, the trigeminal porus was opened following the fibers of the mandibular nerve, and prepontine dura mater was reached following the projection of the anterolateral triangle.

### 3:20 Anatomical Illustration.

This image shows the trajectory of the prepontine dura mater cut in order to enter into the posterior fossa. A cut was made from medial to lateral, starting from a point located between trochlear nerve superiorly and abducens nerve inferiorly reaching the trigeminal porus opening.

### 3:42 Exposure of the Cyst and Brainstem Decompression.

The cystic component of the lesion was identified and evacuated to decompress the brainstem. Neuronavigated aspirator was a useful instrument in complex case for surgical orientation.

### 3:55 Surgical Field Inspection.

Using a 30° endoscope, surgical field was inspected after adequate brainstem decompression. The left abducens nerve and anterior inferior cerebellar artery were identified, as well as the left VII–VIII complex and IX–XI nerve complex.

### 4:19 Resection of the Solid Component.

At this point the solid component of the tumor was identified and removed. Left MEP showed improvement to ensure adequate decompression of the brainstem.

### 4:32 Reconstruction.

The small dural defect is repaired in a multilayered fashion using dural substitute, fibrin glue, and autologous abdominal fat.^5^

### 4:44 Postoperative Neuroimaging and Postoperative Clinical Outcome.

Postoperative MRI on the right, compared with preoperative images, shows significant reduction of the cystic portion of the lesion with a good reduction of brainstem compression.

The patient presented a rapid improvement of the preoperative symptoms and was able to walk unaided with improved neurological function. Stitches of the subbrow wound was removed on day 7 with no complications. There was no cerebrospinal fluid leakage or infection.

There’s no clinically significant recurrence at the brainstem at 3-year follow-up.

## Disclosures

The authors report no conflict of interest concerning the materials or methods used in this study or the findings specified in this publication.

## Author Contributions

Primary surgeon: Mak, Xu. Assistant surgeon: Yuen. Editing and drafting the video and abstract: Paglia, Ng, Xu, Sgarbanti, Corrivetti, de Notaris. Critically revising the work: Mak, Paglia, Ng, Xu, Corrivetti, de Notaris. Reviewed submitted version of the work: Mak, Paglia, Ng, Xu, de Notaris. Approved the final version of the work on behalf of all authors: Mak. Supervision: Mak.

## Supplemental Information

Supplemental Figs. 1-3

### Patient Informed Consent

The necessary patient informed consent was obtained in this study.
