# Supplementary material for: Endoscopic transorbital approach to posterior fossa recurrent craniopharyngioma
Source: Neurosurg Focus Video. 2025 Apr 1;12(2):V3. doi: 10.3171/2025.1.FOCVID24177 (PMC12070329; doi:10.3171/2025.1.FOCVID24177)
Supplement: Supplemental Figs. 1-3 [file SupplementalFigs1-3_FOCVID24-177.pdf]

NEUROSURGICAL FOCUS VIDEO

ONLINE ONLY

Supplemental material

Endoscopic transorbital approach to posterior fossa recurrent craniopharyngioma

Paglia et al.

https://thejns.org/doi/abs/10.3171/2025.1.FOCVID24177

DISCLAIMER The *Journal of Neurosurgery* acknowledges that the following section is published verbatim as submitted by the authors and did not go through either the *Journal's* peer-review or editing process.

© 2025 The authors, CC BY 4.0 (http://creativecommons.org/licenses/by/4.0/)

Supplemental Figure 1

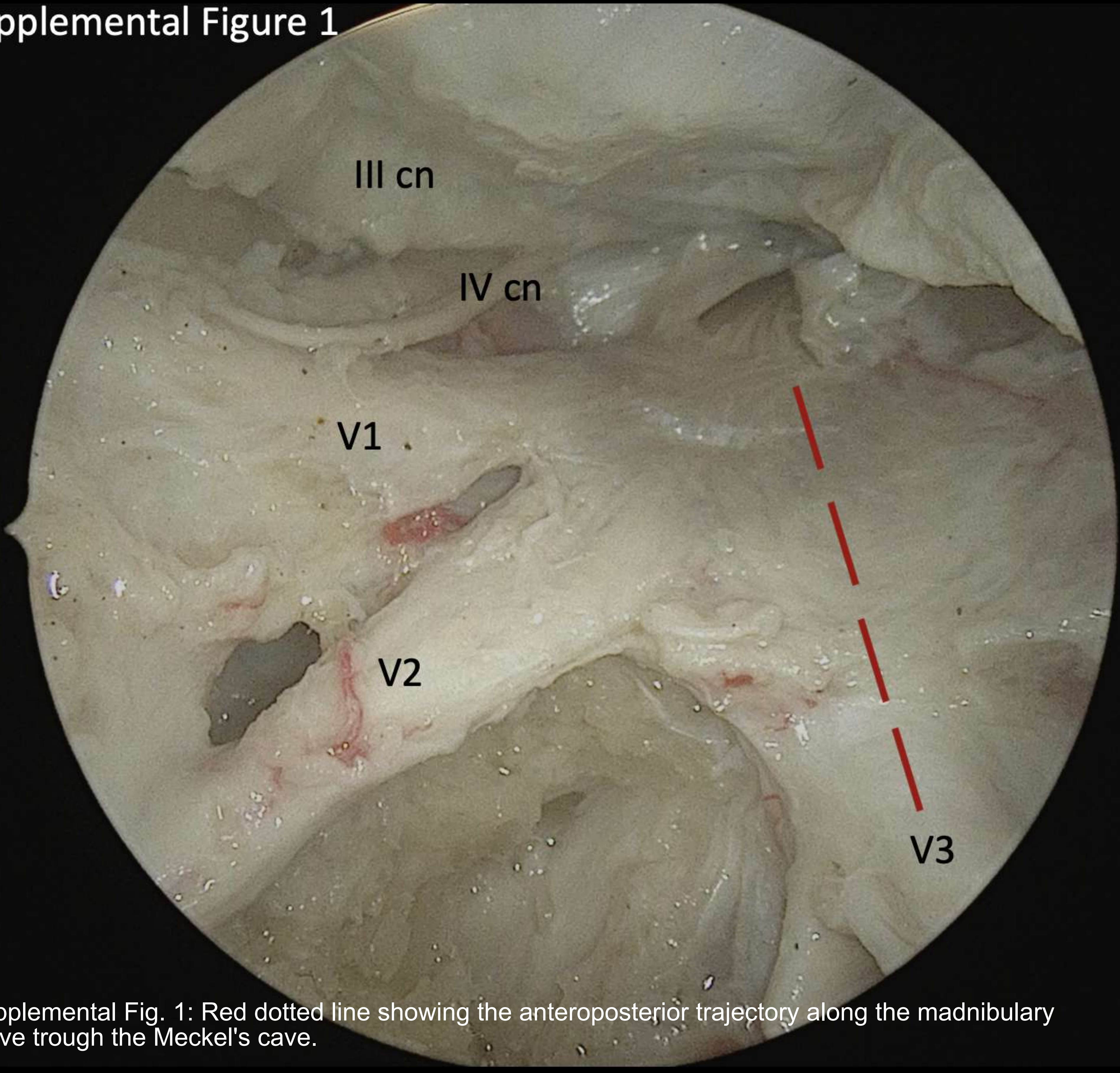

Supplemental Fig. 1: Red dotted line showing the anteroposterior trajectory along the madnibulary nerve trough the Meckel's cave.

Supplemental Figure 2

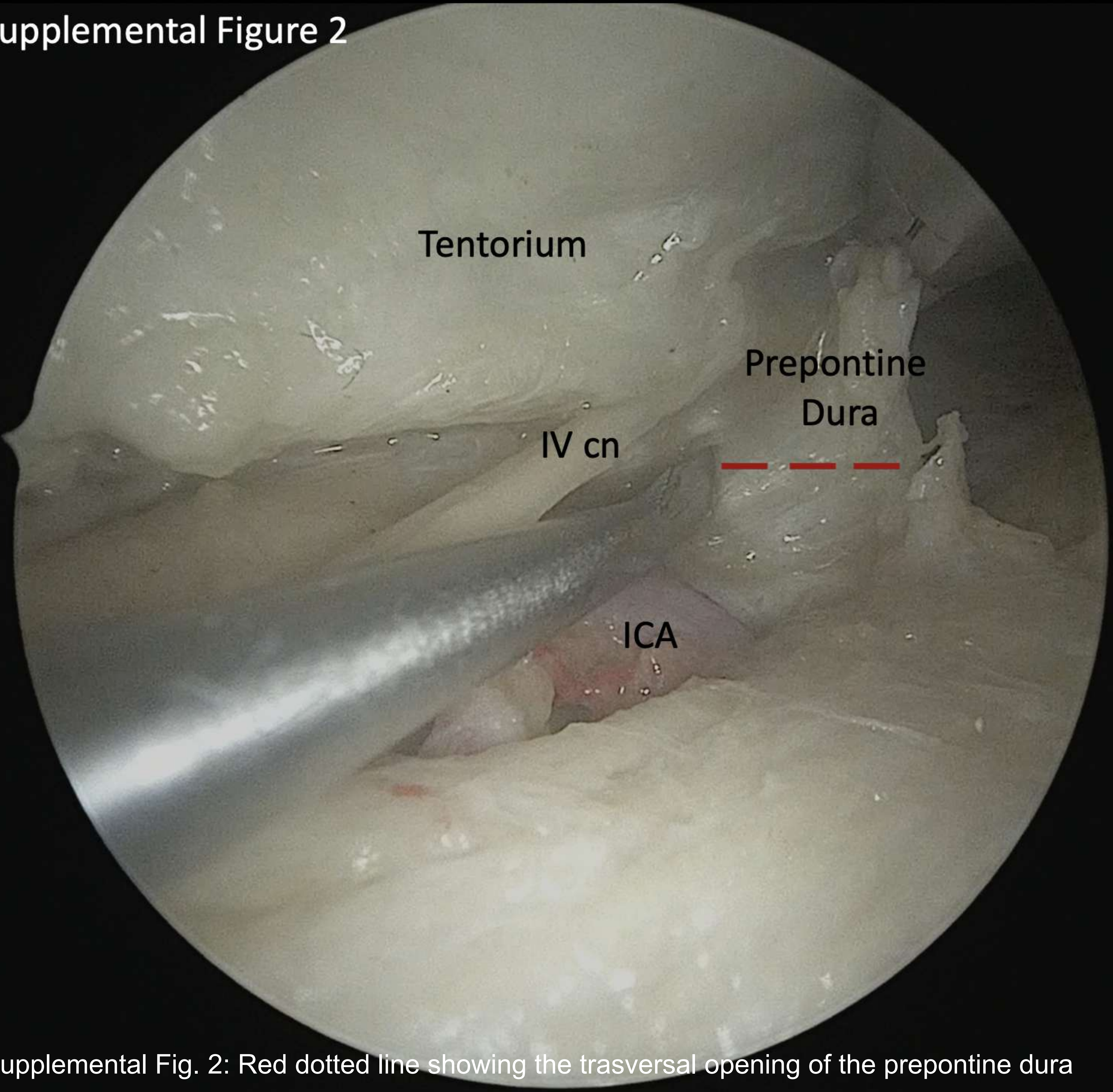

Supplemental Fig. 2: Red dotted line showing the trasversal opening of the prepontine dura

Supplemental Figure 3

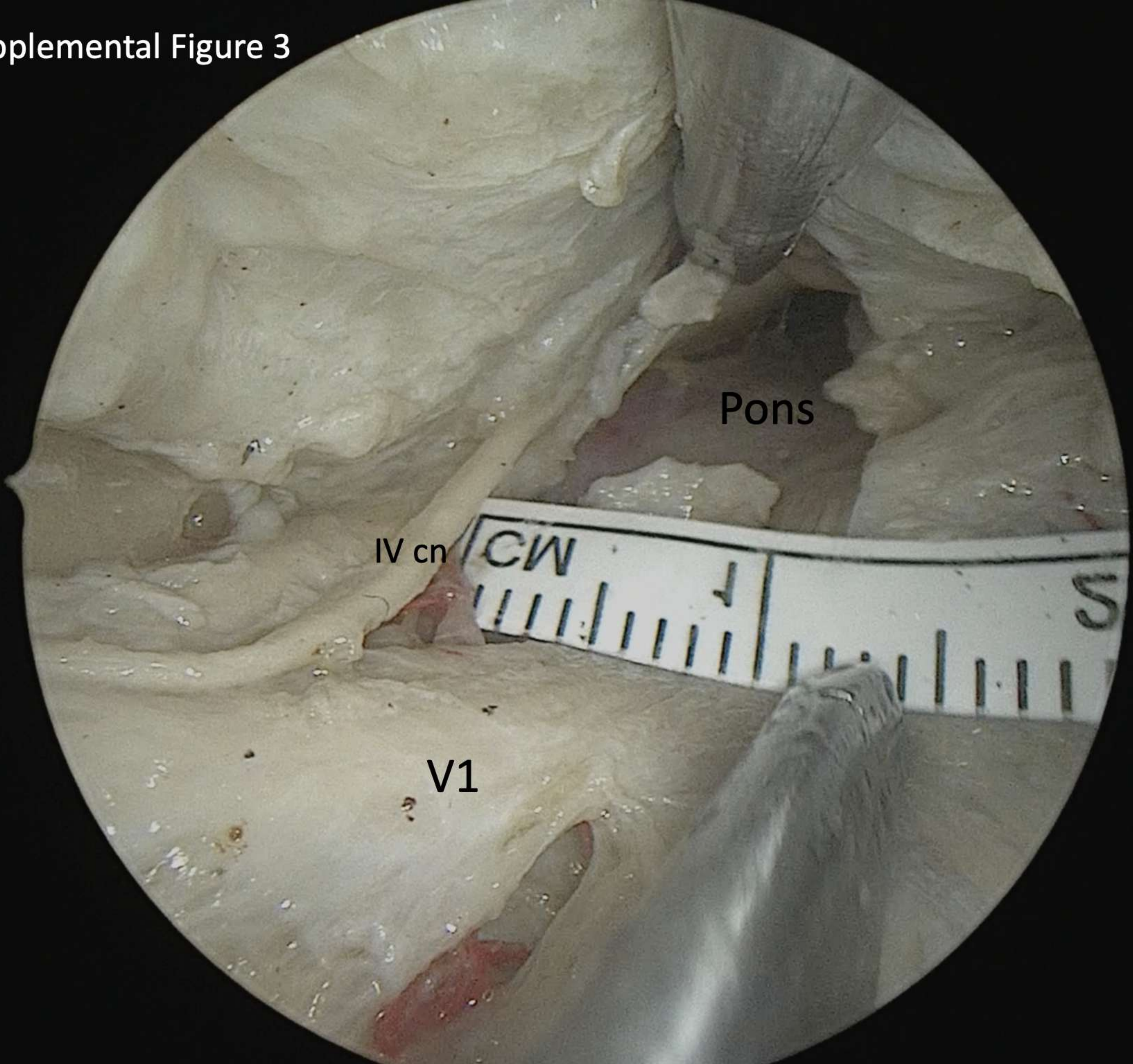

Supplemental Fig. 3: Final opening of the tentorial edge to expose the superolateral portion of the pons
